# Supplementary material for: Deep learning for synthetic microstructure generation in a materials-by-design framework for heterogeneous energetic materials
Source: Sci Rep. 2020 Aug 6;10:13307. doi: 10.1038/s41598-020-70149-0 (PMC7413342; doi:10.1038/s41598-020-70149-0)
Supplement: Supplementary file 1 — Supplementary Information. [file 41598_2020_70149_MOESM1_ESM.pdf]

Deep learning for synthetic microstructure generation in a materials-by-design framework for heterogeneous energetic materials

Sehyun Chun[1], Sidhartha Roy[2], Yen Thi Nguyen[2], Joseph B. Choi[1], H. S. Udaykumar[2,*], and Stephen S. Baek[1,*]

[1]Department of Industrial and Systems Engineering, University of Iowa, Iowa City, IA 52242, United States
[2]Department of Mechanical Engineering, University of Iowa, Iowa City, IA 52242, United States
[*]{hs-kumar, stephen-baek}@uiowa.edu

Extended Data

Additional Microstructure Images

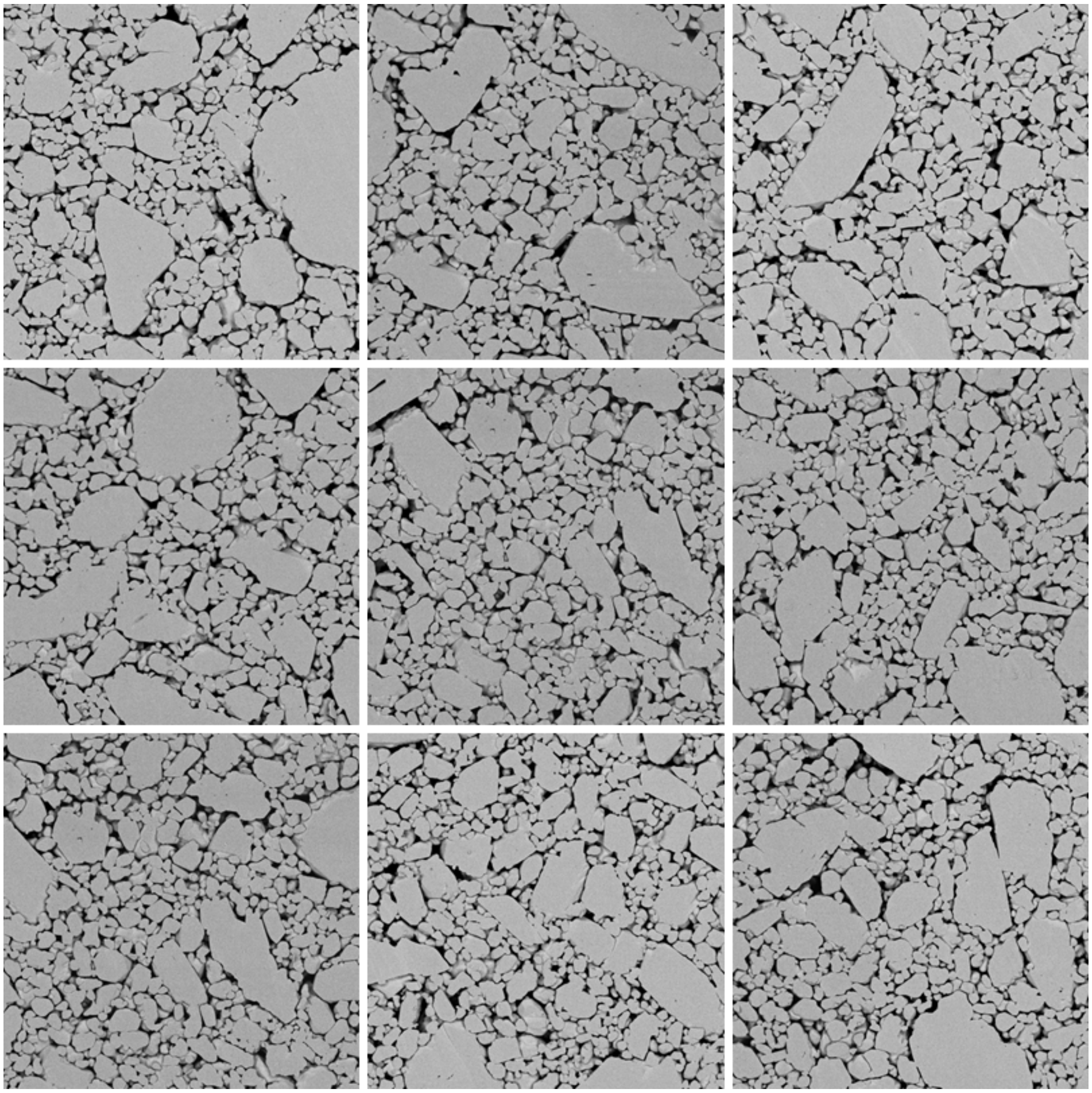

Extended Data Figure 1. Additional RealμS images of cyclotetramethylene-tetranitramine (HMX) pressed energetic material obtained using SEM.

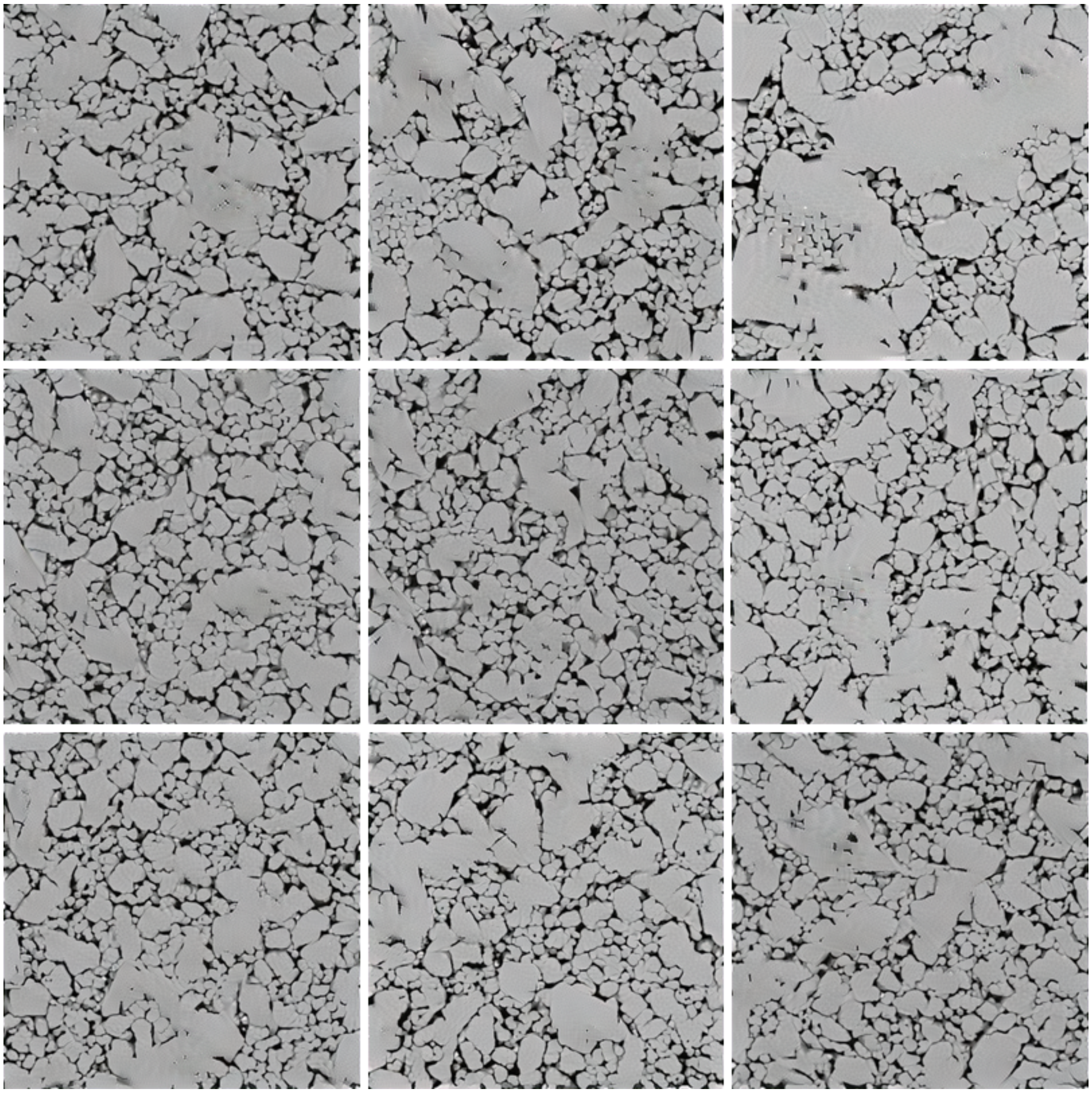

Extended Data Figure 2. Additional TL-generated SynμS images.[1]

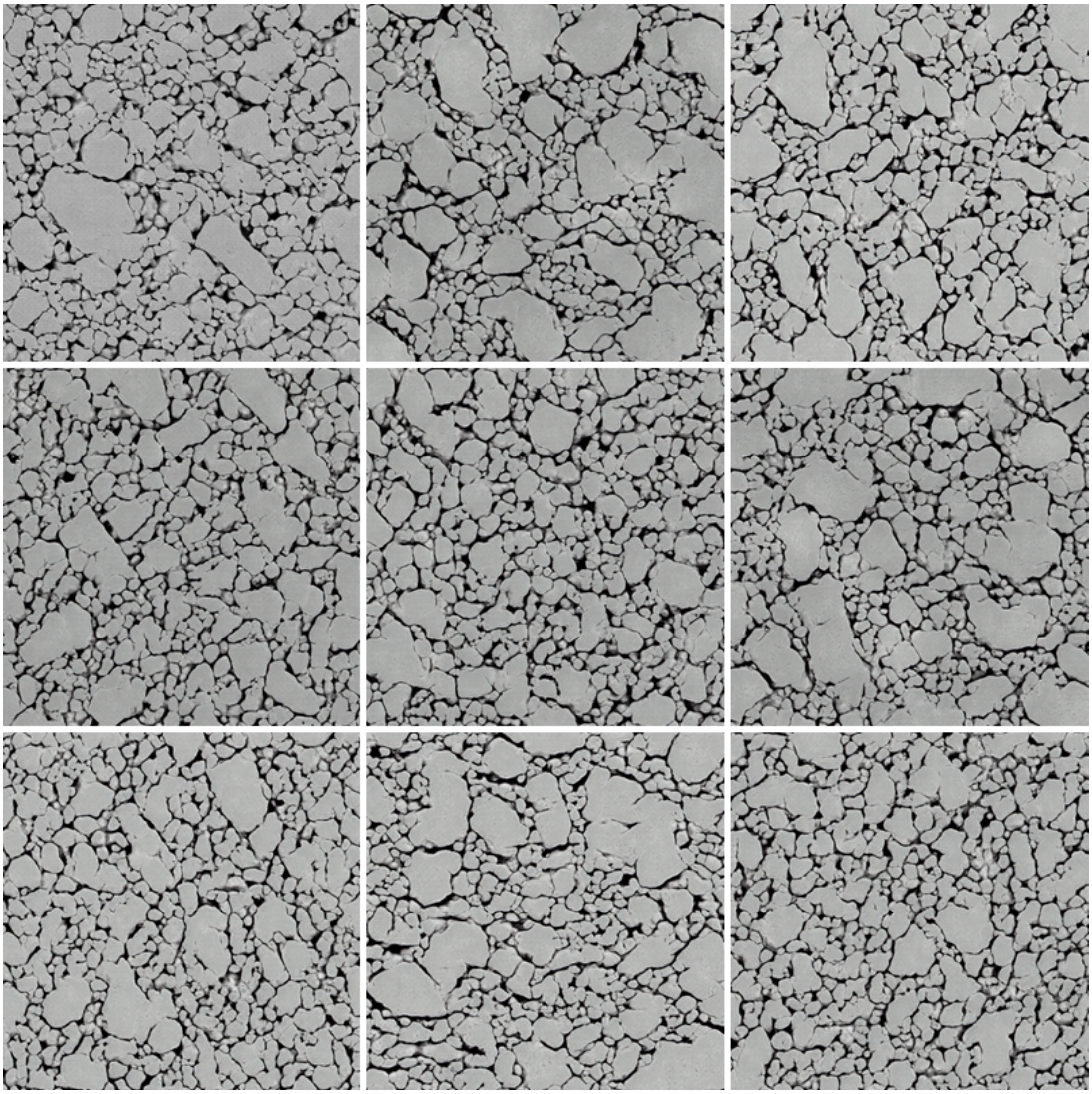

Extended Data Figure 3. Additional GAN-generated SynμS images (ours).

Morphometry of Voids and Crystals

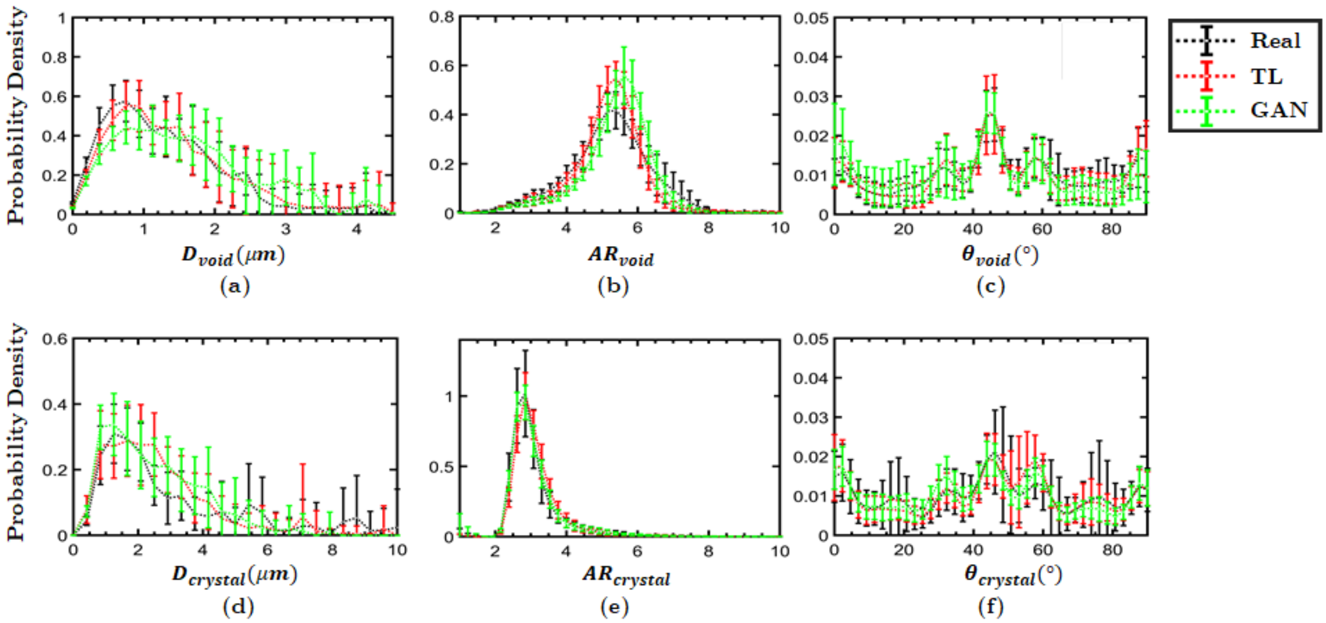

Extended Data Figure 4. Stochastic morphometry of RealμS and SynμS. (a-c) diameter, aspect ratio, and orientation distributions of voids, respectively (identical to the main text Figure 3). (d-f) diameter, aspect ratio, and orientation distributions of crystals, respectively. Both void and crystal morphology show good agreement between RealμS and SynμS.

Correlation between the GAN parameter λ and the morphometric measures

A multivariate linear regression was conducted to illuminate the correlation between each dimension of the GAN parameter λ and the conventional morphometric descriptors including void/crystal diameter ($D_{void/crystal}$), aspect ratio ($AR_{void/cristal}$), and orientation ($\theta_{void/crystal}$). The total of 150 GAN-generated SynμS were drawn randomly by sampling uniform random variables in [-1,1] and use them as input parameters λ and ρ for GAN. Mean void/crystal diameter, aspect ratio, and orientation were computed for each sample and used for the correlation analysis. Outliers for each of the morphometric descriptors were removed using Cook's distance[2] with one-fourth of the sample size, or 0.026, as a threshold and observations above the threshold were considered as an outlier. The mean and the 95% confidence bands of the regression coefficients across 100 bootstrap experiments are displayed in Extended Data Figures 5 and 6 and the values are reported in Extended Data Tables 1 and 2. Certain GAN parameters λ are significantly correlated with each of the morphometry parameters. For example, λ_2 exhibits a strong positive correlation with the void diameter ($p < 0.001$) so that an increase of the λ_2 value would result larger void diameters in the SynμS. Similarly, λ_3, λ_7, λ_{10}, and λ_{12} exhibit strong positive correlations with the crystal diameter ($p < 0.001$) such that an increase of these GAN parameters would produce larger crystals.

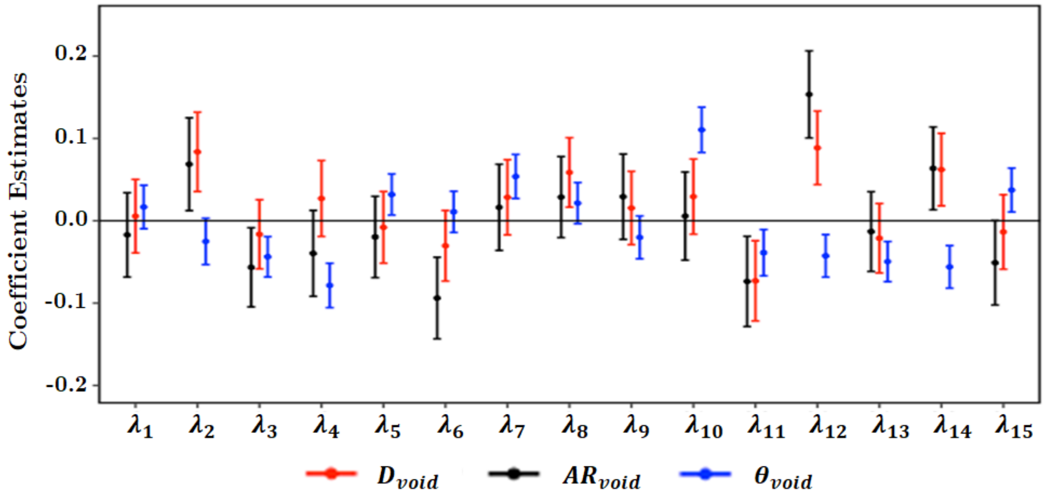

Extended Data Figure 5. Estimated coefficients of the GAN parameters on the void morphometry and their 95% confidence bands.

λ	Estimated Coefficients			Standard Errors		
	D_{void}	θ_{void}	AR_{void}	D_{void}	θ_{void}	AR_{void}
Intercept	0.5054	0.4323	0.5097	0.0126	0.0074	0.0148
λ_1	0.0037	0.0157	-0.0139	0.0226	0.0133	0.0259
λ_2	0.0845	-0.0232	0.0719	0.0244^{***}	0.0142	0.0285
λ_3	-0.0167	-0.0232	0.0719	0.0212	0.0124^{*}	0.0243
λ_4	0.0233	-0.0777	-0.0368	0.0233	0.0136^{***}	0.0264
λ_5	-0.0099	0.0314	-0.0170	0.0220	0.0126	0.0249
λ_6	-0.0316	0.0123	-0.0934	0.0217	0.0126	0.0250^{**}
λ_7	0.0319	0.0534	0.0188	0.0231	0.0135^{**}	0.0264
λ_8	0.0632	0.0211	0.0274	0.0213^{*}	0.0126	0.0249
λ_9	0.0131	-0.0227	0.0392	0.0225	0.0131	0.0262
λ_{10}	0.0274	0.1104	0.0088	0.0231	0.0139^{***}	0.0271
λ_{11}	-0.0714	-0.0390	-0.0777	0.0246^{*}	0.0142	0.0277^{*}
λ_{12}	0.0883	-0.0425	0.1502	0.0226^{**}	0.0131^{*}	0.0267^{***}
λ_{13}	-0.0196	-0.0479	-0.0123	0.0213	0.0123^{**}	0.0245
λ_{14}	0.0652	-0.0559	0.0639	0.0222^{*}	0.0131^{**}	0.0254
λ_{15}	-0.0107	0.0388	-0.0544	0.0229	0.0135^{*}	0.0259
$\bar{R}^2$	0.3269	0.5906	0.4209			
F-statistic vs. Constant Model	5.769	14.92	7.990			
p-value	$2.39e{-5}$	$6.92e{-13}$	$3.90e{-7}$			
N	142	138	139			

$p < 0.001(***), 0.01(**), 0.05(*)$

Extended Data Table 1. The association between the GAN parameters and the void morphometry.

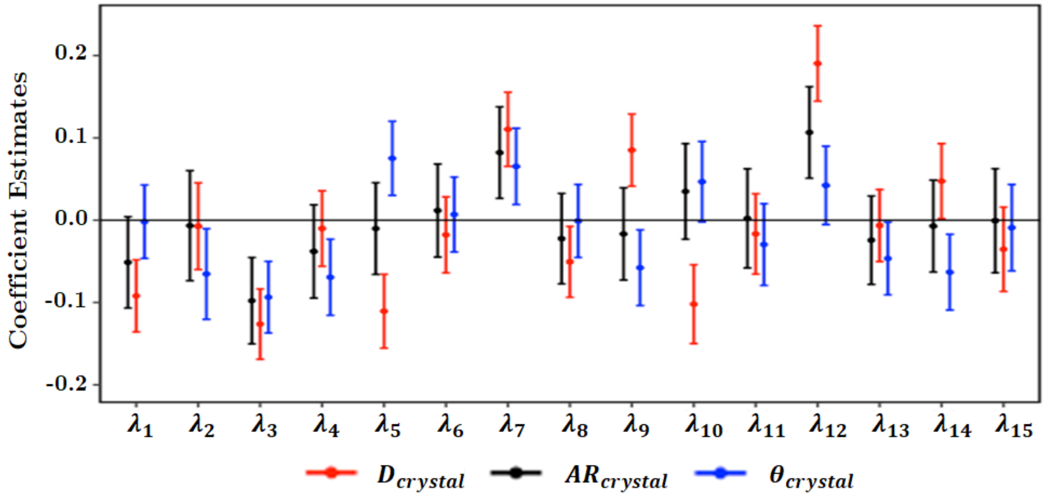

Extended Data Figure 6. Estimated coefficients of the GAN parameters on the crystal morphometry and their 95% confidence bands.

λ	Estimated Coefficients			Standard Errors		
	D_{crystal}	θ_{crystal}	AR_{crystal}	D_{crystal}	θ_{crystal}	AR_{crystal}
Intercept	0.2017	0.4045	0.2841	0.0128	0.0131	0.0159
λ_1	-0.0933	-0.0055	-0.0506	0.0218**	0.0224	0.0274
λ_2	-0.0111	-0.0710	-0.0056	0.0268	0.0283*	0.0331
λ_3	-0.1206	-0.0947	-0.1008	0.0214***	0.0215**	0.0265*
λ_4	-0.0081	-0.0698	-0.0407	0.0229	0.0232*	0.0283
λ_5	-0.1129	0.0692	-0.0128	0.0221**	0.0224*	0.0273
λ_6	-0.0184	0.0064	0.0154	0.0229	0.0226	0.0283
λ_7	0.1104	0.0649	0.0846	0.0221***	0.0230	0.0275*
λ_8	-0.0479	-0.0020	-0.0240	0.0217	0.0221	0.0268
λ_9	0.0877	-0.0580	-0.0156	0.0222**	0.0227	0.0280
λ_{10}	-0.1024	0.0516	0.0328	0.0240***	0.0245	0.0290
λ_{11}	-0.0175	-0.0298	0.0017	0.0242	0.0249	0.0296
λ_{12}	0.1899	0.0404	0.1056	0.0226***	0.0238	0.0278*
λ_{13}	-0.0028	-0.0457	-0.0261	0.0218	0.0219	0.0265
λ_{14}	0.0508	-0.0641	-0.0017	0.0225	0.0228*	0.0279
λ_{15}	-0.0309	0.0053	-0.0008	0.02561	0.02623	0.0313
$\bar{R}^2$	0.6732	0.4068	0.2931			
F-statistic vs. Constant Model	18.71	6.993	4.582			
p-value	1.10e−18	1.04e−5	1.22e−4			
N	127	126	127			

$p < 0.001(***), 0.01(**), 0.05(*)$

Extended Data Table 2. The association between the GAN parameters and the crystal morphometry.

Spatial Control of the Morphology

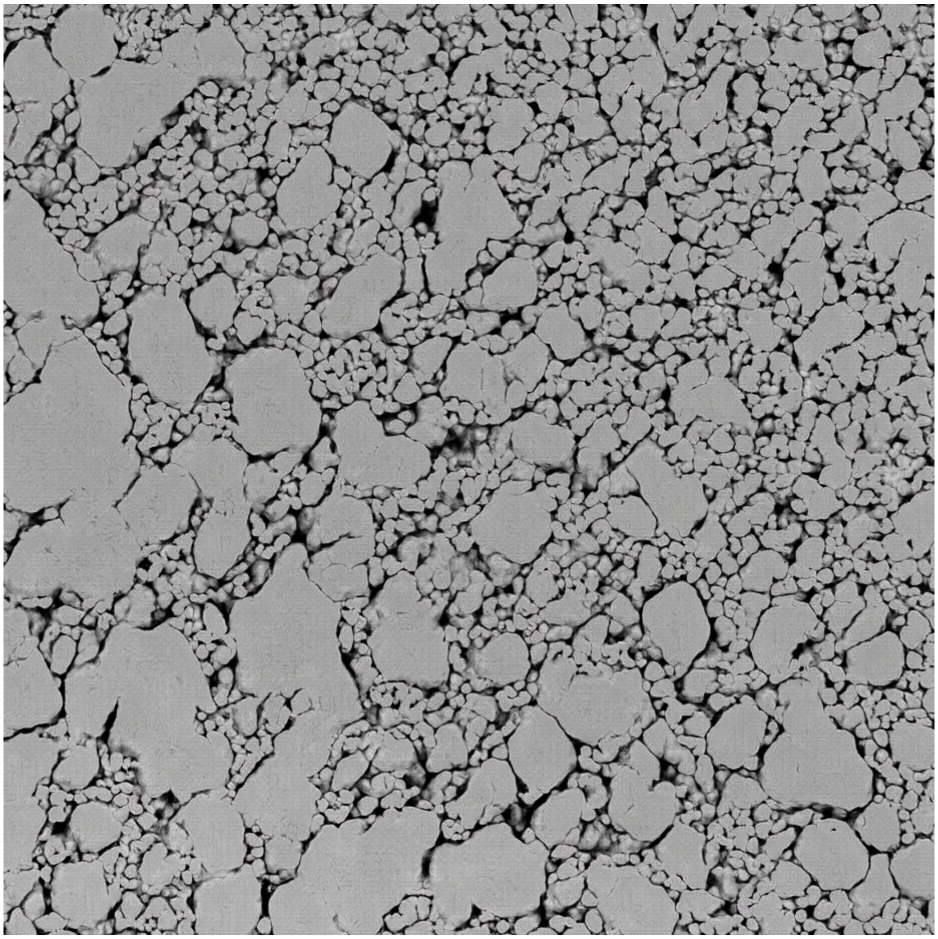

Extended Data Figure 7. Bi-linear interpolation of the GAN parameters.

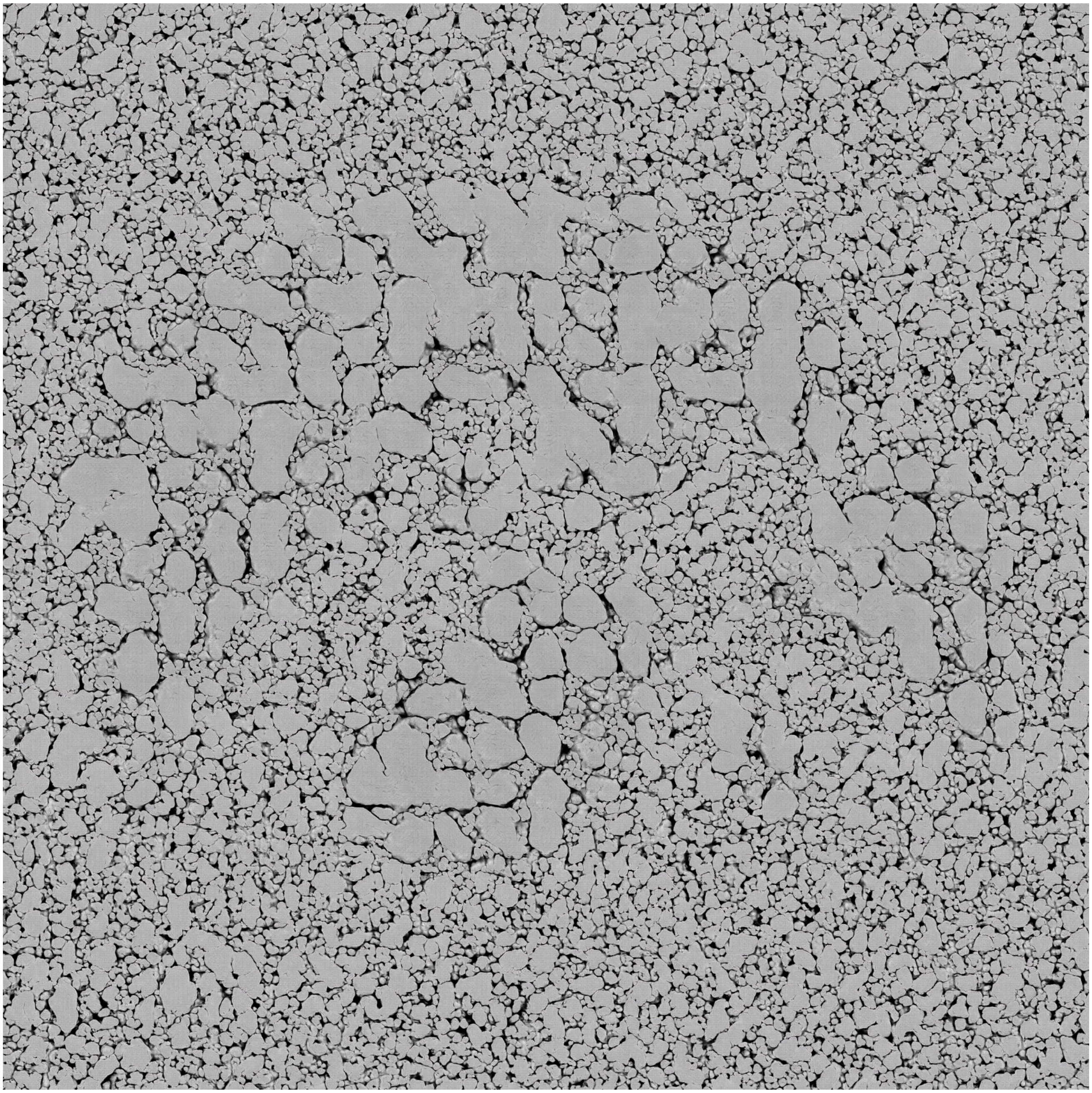

Extended Data Figure 8. High-resolution display of Figure 12(b).

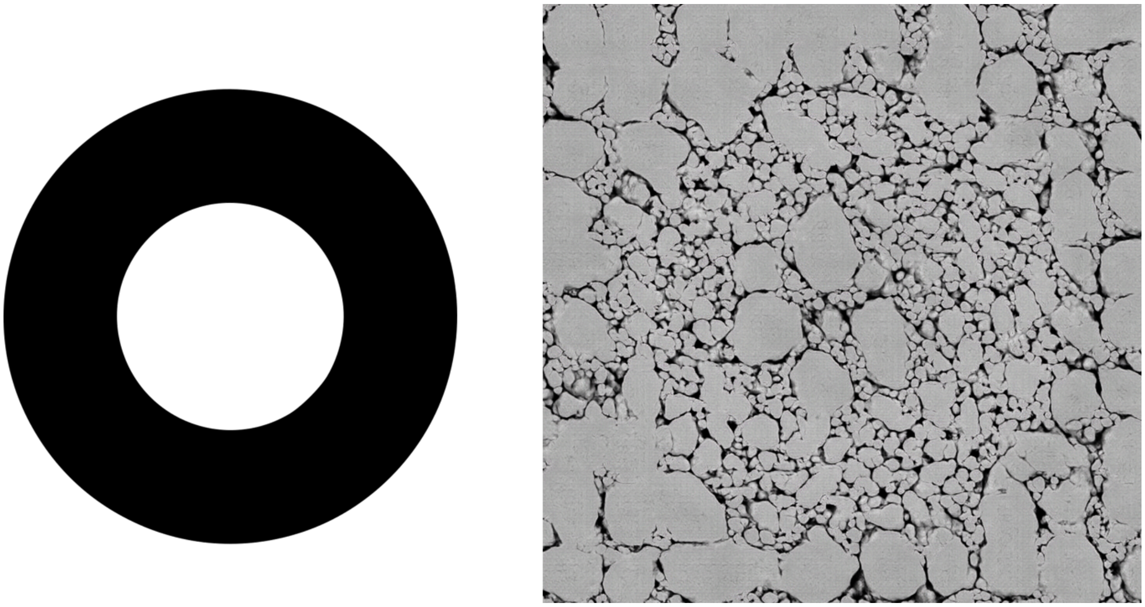

Extended Data Figure 9. A layout of global morphology parameters (left) and the corresponding GAN-SynμS (right). Similar to main text Figure 12 (b), white background regions are painted with large crystal morphology parameter and foreground regions are painted with small crystal morphology parameter.

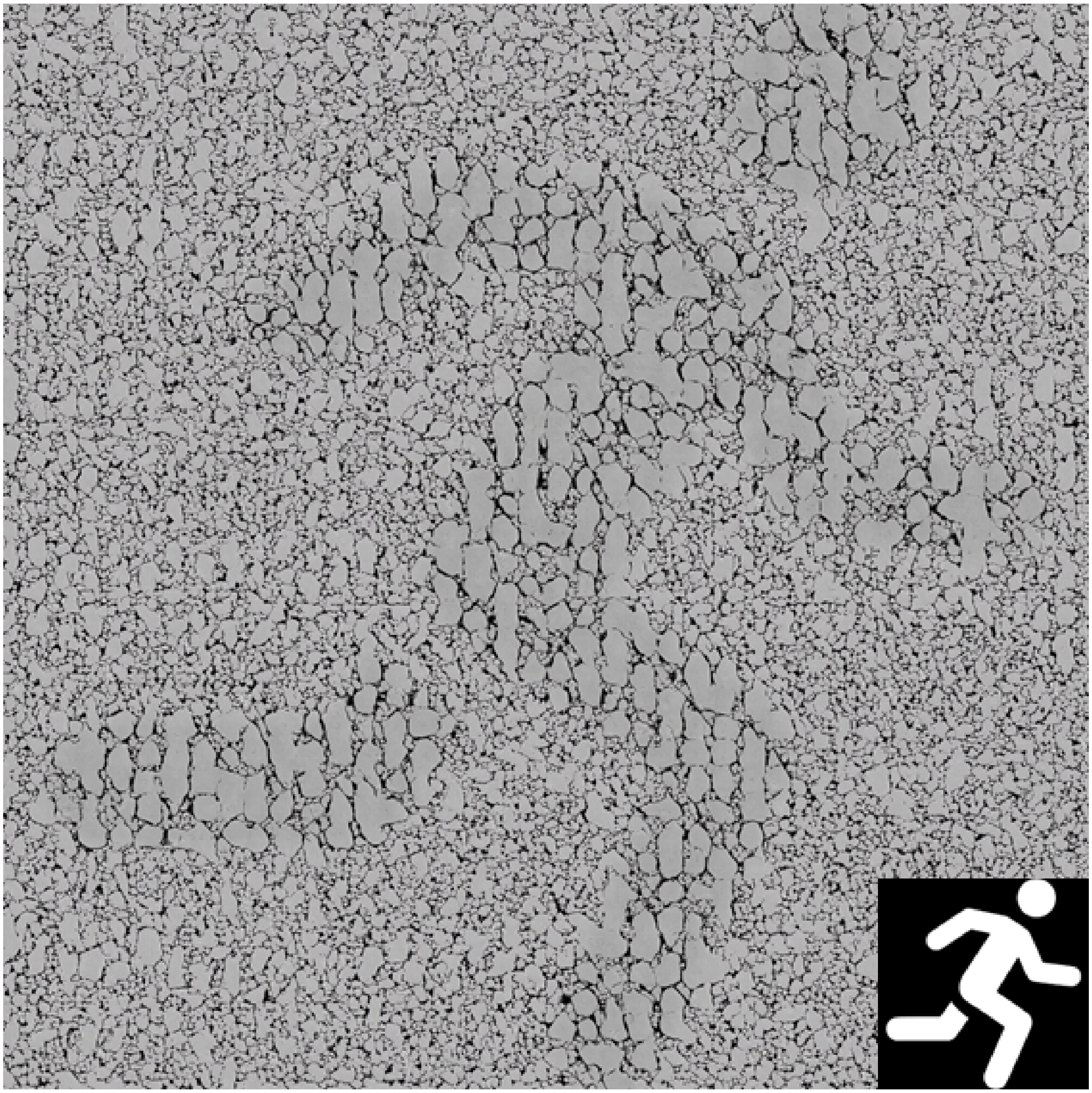

Extended Data Figure 10. A layout of global morphology parameters (bottom right corner) and the corresponding GAN-SynμS. Black background regions are painted with small crystal morphology parameter and white pixel regions are painted with large crystal morphology parameter.

References

1. Li, X. *et al.* A transfer learning approach for microstructure reconstruction and structure-property predictions. *Sci. Reports* **8**, DOI: 10.1038/s41598-018-31571-7 (2018).

2. Cook, R. D. Detection of influential observation in linear regression. *Technometrics* **42**, 65–68 (2000).
